# Role of Ectopic Olfactory Receptors in the Regulation of the Cardiovascular–Kidney–Metabolic Axis

**DOI:** 10.3390/life14050548

**Published:** 2024-04-25

**Authors:** Mitchell R. Beito, Sadia Ashraf, Dorcas Odogwu, Romain Harmancey

**Affiliations:** Division of Cardiology, Department of Internal Medicine, McGovern Medical School, The University of Texas Health Science Center at Houston, Houston, TX 77030, USA; mitchell.r.beito@uth.tmc.edu (M.R.B.); sadia.ashraf@uth.tmc.edu (S.A.); dorcas.odogwu@uth.tmc.edu (D.O.)

**Keywords:** G protein-coupled receptor, olfactory receptor, cardiovascular, kidney, metabolism, heart disease

## Abstract

Olfactory receptors (ORs) represent one of the largest yet least investigated families of G protein-coupled receptors in mammals. While initially believed to be functionally restricted to the detection and integration of odors at the olfactory epithelium, accumulating evidence points to a critical role for ectopically expressed ORs in the regulation of cellular homeostasis in extranasal tissues. This review aims to summarize the current state of knowledge on the expression and physiological functions of ectopic ORs in the cardiovascular system, kidneys, and primary metabolic organs and emphasizes how altered ectopic OR signaling in those tissues may impact cardiovascular–kidney–metabolic health.

## 1. Introduction

G protein-coupled receptors (GPCRs) constitute the largest family of plasma membrane receptors in eukaryotes. These receptors interact with a diverse array of ligands, including photons, ions, hormones, and neurotransmitters to rapidly adapt cellular functions to shifting environmental conditions. The omnipresence of their cellular signaling in the regulation of mammalian physiology, coupled with a high ‘druggability’, has long made GPCRs a therapeutic target of choice for the treatment of many human diseases [1,2,3]. Pharmacological modulation of GPCR signaling is particularly relevant to the cardiovascular system, for which many GPCR agonists and antagonists have become the standard of care for several acute and chronic conditions such as hypertension, coronary artery disease, and heart failure [4]. In fact, nearly a third of the drugs used for the treatment of cardiovascular disease target GPCRs, with modulators of the adrenergic and angiotensin receptors representing the bulk of the current therapeutic arsenal [5]. In spite of these medical breakthroughs, cardiovascular disease remains a leading cause of morbidity and mortality worldwide, and new pharmacological approaches targeting alternative candidate GPCR pathways are constantly being investigated [6].

The recent uptick in the cardiovascular disease death rate, fueled in part by the growing epidemics of obesity and diabetes, has made the need for better therapeutic strategies more urgent [7,8,9]. Poor cardiovascular–kidney–metabolic health, which reflects the pathophysiological interactions between metabolic risk factors, chronic kidney disease (CKD), and the cardiovascular system, is increasingly recognized as the main driving force behind the exacerbation of cardiovascular disease, particularly among young individuals [10,11]. There are more than 1200 GPCRs expressed in the human genome, among which fewer than half have been investigated as receptors for endogenously produced molecules such as neurotransmitters and hormones. However, with the discovery that chemosensory GPCRs, that is, receptors recognizing chemical signals of odorants and tastants in the environment, are functionally expressed throughout the body came the realization that hundreds more GPCRs may actually contribute to the regulation of physiological processes [12,13,14]. Therefore, these chemosensory GPCRs represent a vast untapped potential for the identification of druggable mechanisms that may be targeted to prevent and even possibly revert the development of cardiovascular–renal disorders linked to the impairment of metabolic homeostasis.

Olfactory receptors (ORs), also known as odorant receptors, are class A GPCRs expressed in the olfactory sensory neurons of the nasal epithelium [15]. Since the initial discovery of their critical role in the neural coding of the sense of smell, and thanks to advances in global gene expression profiling techniques, it has become increasingly clear that many ORs are also expressed in non-olfactory tissues [16,17]. Over the past decade, investigations from a growing number of research teams have uncovered the extranasal functions of several of these so-called ectopic ORs, including in metabolic tissues, the kidneys, and most recently, the heart and vessels. The present article aims to provide an overview of the main OR signaling features in mammals and to summarize the current state of knowledge about the physiological processes regulated by ectopic ORs in the cardiovascular and renal systems, with a particular emphasis on the OR-mediated mechanisms that may contribute to the pathogenesis of cardiovascular disease and CKD. Additionally, we review the emerging molecular mechanisms linking ectopic OR activity to the control of metabolic homeostasis and the potential impact on cardiovascular–kidney–metabolic health, with the hope it will stimulate research in the area and increase the likelihood of developing novel therapies for the better management of cardiovascular disease.

## 2. Characterization of Mammalian Olfactory Receptors

### 2.1. Discovery of Olfactory Receptors in the Nasal Epithelium

In 1991, Linda Buck and Richard Axel cloned and characterized the first 18 members of the OR gene superfamily. Their identification of odorant receptors was based on prior findings that such receptors were likely coupled to GTP-binding proteins, on the assumption that they would exhibit a wide diversity to allow the sensory integration of thousands of distinct odorous molecules, and most importantly that their expression should be restricted to the olfactory neuroepithelium. Buck and Axel’s molecular strategy combining the degenerate PCR amplification of GPCR homologs from rat olfactory epithelium RNA, the digestion of the PCR products by restriction endonuclease, and the verification of tissue expression specificity by Northern blot led to their discovery of a multigene family fulfilling all three criteria [18]. In the years that followed this initial discovery, sequence alignments and structure–function analyses established both the conserved and unique features that define odorant receptors amongst the GPCR superfamily. Members of the OR subfamily retain the signature snake-like, seven-transmembrane domain topology, with conserved proline residues within the fourth to seventh domains introducing kinks in the α-helices, and an aspartate–arginine–tyrosine triplet in the third transmembrane domain that is critical for signal transduction [19]. But in stark contrast with other GPCR subfamilies, ORs display strong hypervariability in their third, fourth, and fifth transmembrane domains. This hypervariability is thought to provide the specificity for the types of odorants that can bind to each receptor [20,21]. However, ORs usually exhibit a rather loose specificity toward ligands, so that one odorant molecule can interact with several different ORs with varying affinities, and one OR can be activated by a range of different odorants [20,22]. This loose ligand specificity is the basis of a molecular coding scheme allowing the mammalian olfactory system to rely only on a few hundred ORs to discriminate several thousand odorants [23]. There are about 400 and 1000 functional OR genes in humans and mice, respectively, with current statistical models suggesting the numbers may be a direct predictor of olfactory sensitivity and discrimination performance among mammals [24,25]. Whether the extranasal tissues expressing ectopic ORs employ a similar combinatorial receptor coding scheme to finely tune cellular response is currently unknown.

### 2.2. Deorphanization of Olfactory Receptors

The odor molecules recognized by ORs are structurally very diverse and include alcohols, aldehydes, ketones, carboxylic acids, sulfur-containing compounds, and essential oils [26,27]. While the lack of X-ray crystal structures for ORs has hampered early attempts at deorphanizing these receptors, a breakthrough in OR–ligand pairing came with the discovery of the receptor-transporting protein (RTP) family, and most particularly, RTP1 and RTP2. The proteins RTP1 and RTP2 are expressed in olfactory neurons to promote both the translocation and the functionality of ORs at the plasma membrane [28]. Similar, though weaker, pro-olfactory signaling effects are obtained with the expression of a facilitator of endoplasmic reticulum–mitochondria interactions known as receptor expression-enhancing protein 1 (REEP1) [28,29]. Exploiting the functions of these proteins has allowed for the heterologous expression of ORs in cell models suitable for ligand screening [30]. The subsequent addition of in silico screening methodologies to in vitro validation approaches has immensely contributed to the identification of both natural and synthetic OR ligands [31,32,33]. New advances in molecular modeling and the development of artificial intelligence-driven prediction tools will undoubtedly accelerate OR–ligand pairing efforts [34,35]. Recent estimates suggest that less than 50% of human ORs and 20% of murine ORs have been deorphanized [34]. The Molecule to Olfactory Receptor Database (M2OR) is one of the most recently introduced online tools that provides curated data from more than 75,000 bioassay experiments to allow for easy exploration of the current state of the research on OR–ligand pairing [36].

The odorant molecules, flavor compounds, and metabolites that constitute the bulk of ectopic OR ligands may enter the body via inhalation, enterally, or trans-dermally. From there, they can reach the bloodstream and virtually any cells and tissues within the organism [37]. While the absorption kinetics and metabolism of these molecules will ultimately depend on the uptake route, their release from food components and passage through the gastrointestinal tract is regarded as the principal source of ligands for ectopically expressed ORs [37,38]. Dozens of exogenous natural and synthetic odorant and flavor compounds originating from our diet have already been identified as key modulators of OR function [39]. Most importantly, compounds derived from the metabolic activity of the gut microbiota such as short-chain fatty acids have been shown to signal through ORs expressed in the brain and kidney [40,41]. These findings highlight yet another way by which dietary habits and the gut microbiome may contribute to the regulation of physiological functions in health and disease.

### 2.3. Olfactory Receptor Signaling Pathways

Even prior to their identification, experimental evidence pointed toward ORs employing the classical pathway of seven-transmembrane receptor signaling. In the mid to late 1980s, the exposure of cilia isolated from frog and rat olfactory epitheliums to various odorants was found to cause an elevation in intracellular cAMP levels that was dependent on the presence of GTP and hence, was likely to be mediated via heterotrimeric G protein activation [42,43]. In vertebrates, ORs signal through the specialized G α subunit G_olf_, and loss of G_olf_ in mice results in a significant reduction in the electrophysiological response to odors [44]. Activated G_olf_ subsequently stimulates the type 3 adenylyl cyclase (AC3), which leads to cAMP-mediated membrane depolarization through recruitment of a calcium (Ca^2+^)-permeable cyclic nucleotide-gated (CNG) channel that has also been linked to general anosmia when inactivated in the mouse [45,46]. Besides this canonical olfactory signaling pathway, some olfactory sensory neurons have also been reported to use cGMP and inositol 1,4,5-triphosphate (IP3) as alternative intracellular messengers [47,48]. Although G_olf_ and AC3 are predominantly expressed in cilia, these components of the olfactory signaling pathway have also been detected in a variety of cells and tissues including smooth muscle cells, the heart, and kidneys, thus supporting the existence of shared cAMP-mediated intracellular mechanisms for ectopically expressed ORs [49,50,51]. Signaling in non-olfactory tissues is also quite diverse and may involve IP3 generation and a rise in intracellular Ca^2+^ levels, or it may even occur independently from heterotrimeric G protein activation depending on the cellular system considered [39,52,53]. The functions of ectopic ORs are as versatile as their signaling mechanisms. Through post-translational enzyme modifications and the regulation of gene expression programs, ectopic ORs have been linked to the modulation of many essential cellular processes such as metabolism, the release of hormones and cytokines, apoptosis, growth, and proliferation [52,53].

### 2.4. Classification and Nomenclature of Olfactory Receptors in Vertebrates

The OR gene family of vertebrates has evolved through a birth-and-death evolution process, in which new genes are created through duplication and are either maintained functional in the genome or eventually become inactive through deletion or other detrimental mutational events [54]. Phylogenetic analyses have distinguished two different types of OR genes as follows: Class I genes, which are associated with the detection of water-soluble molecules, and Class II genes, which are dedicated to the detection of airborne odorants [55]. While being evolutionarily related to fish and other aquatic species, more than 100 of the Class I genes are still functional in mammals, suggesting a well-conserved role for some of those ancestral OR genes [55]. With more than 1000 genes widely distributed as clusters on most chromosomes and covering about 1% of the functional genome, ORs represent the broadest gene family in mammals [56]. The largest repertoires of functional OR genes have been described in the rat (*n* = 1284) and the mouse (*n* = 1194), while primates including humans (*n* = 387) retain a smaller proportion of functional ORs, which coincides with the development of trichromatic color vision as the species’ main sensory feature [57,58].

Sequence similarity classification led to the full repertoire of human OR genes and pseudogenes being divided into 18 families and 300 subfamilies. According to this classification system, gene symbols are composed of the prefix ‘OR’, followed by a family numeral, subfamily letter(s), and a number designating the individual gene within the subfamily considered. Pseudogenes are further identified by the letter ‘P’ at the end of the gene symbol [59,60,61,62]. Different nomenclature systems were initially adopted in some other vertebrate species, including mice and rats, and it was only very recently that efforts were made to assign a unified, human-centric nomenclature based on inter-species hierarchical pairwise similarities [61]. Nomenclature systems previously used to identify OR genes in rodents included the use of the root ‘MOR’ or ‘Olfr’ for the mouse and ‘RnOR’ or ‘Olr’ for the rat, with ‘P’ or ‘-ps’ subsequently added at the end to denote a pseudogene [61,63,64]. The prefix ‘Olfr’ was long used as the official nomenclature system by the Mouse Genome Informatics (MGI) database and has been widely employed in the literature until now. Therefore, we will still use this ‘old’ nomenclature system when referring to mouse ORs below, although names of human orthologs will also be provided when available.

## 3. Olfactory Receptors in the Cardiovascular System

### 3.1. Regulation of Cardiac Function

**Figure 1:**
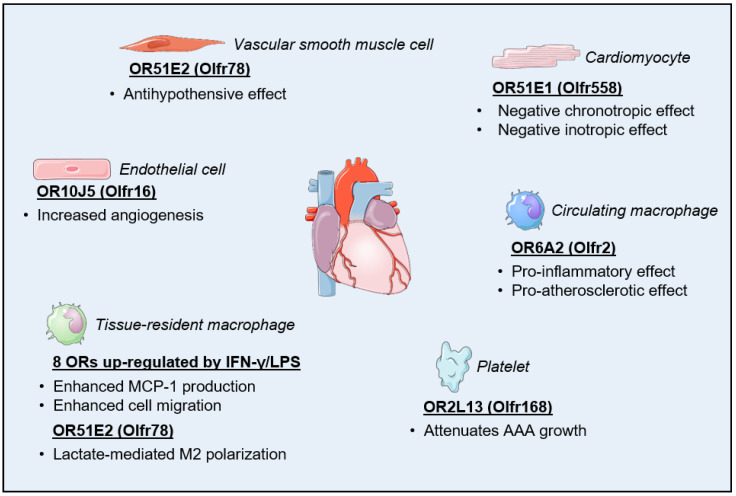
Biological effects of ectopic olfactory receptors expressed in cells of the cardiovascular system. AAA, abdominal aortic aneurysm; MCP-1, monocyte chemoattractant protein-1.

As with many other organs and tissues, the realization that the human heart may express ORs came with the advent of microarray-based, large-scale transcriptome analytical methods [65,66]. By re-analyzing the available human RNA-Seq data from the Illumina Body Map project 2.0, Flegel and colleagues were able to confirm significant cardiac expression not only for more than 20 OR genes and pseudogenes but also for major components of the OR cell trafficking and signaling pathways including G_olf_, AC3, and REEP1 [67]. In a milestone study published in 2017, Jovancevic et al. provided the first, and so far, only evidence for the presence of a functional OR in the heart. Using human-induced pluripotent stem cell-derived cardiomyocytes (hIPSC-CM) and human myocardial tissue slices, the investigators were able to demonstrate that the expression of OR51E1 (Olfr558) at the cardiomyocyte sarcolemma induced negative chronotropic and inotropic effects when stimulated with its known agonist nonanoic acid. The team also identified more than a dozen new agonists for OR51E1 amongst short- and medium-chain fatty acids, some of which may originate from dietary fat or adipose tissue metabolism, that were present at activating concentrations in the plasma of both diabetic and non-diabetic individuals [68]. Although additional in vivo investigations will definitely be needed to establish the physiological relevance of these findings, their study exposes a novel mechanism by which energy metabolism may regulate the mechanical and electrical function of the heart (Figure 1).

**Figure 2:**
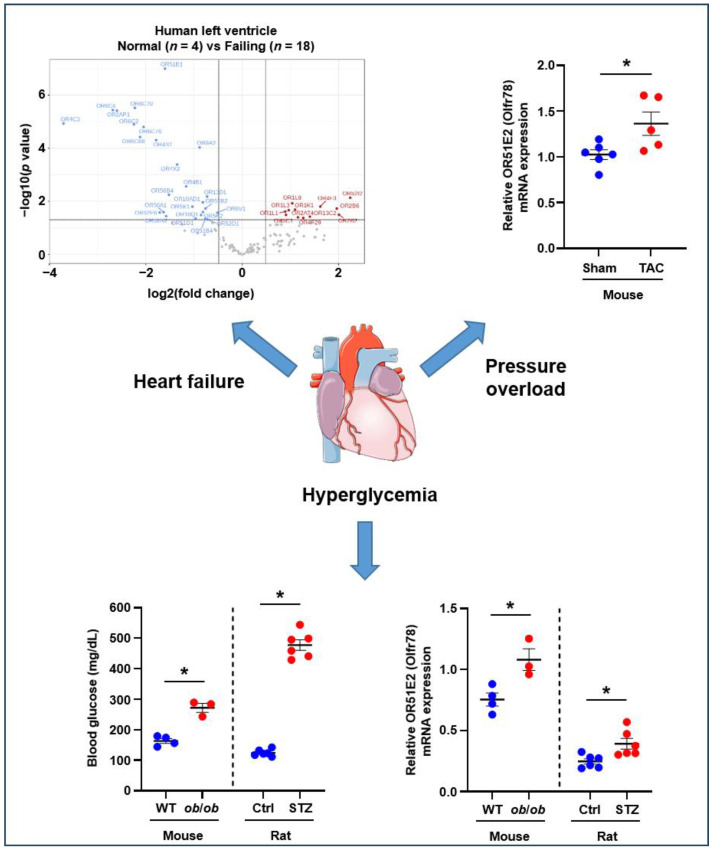
Cardiovascular and metabolic disorders modify cardiac olfactory receptor expression. Clockwise from left to right: qPCR array screening revealed that 38 OR genes are differentially regulated in the human left ventricle in response to heart failure caused by ischemic heart disease; see ref. [71] for details. Up-regulation of OR51E2 (Olfr78) in the left ventricles of C57BL/6 male mice subjected to 21 days of pressure overload induced by transverse aortic constriction (TAC); * *p* < 0.05. Up-regulation of OR51E2 (Olfr78) in the hearts of hyperglycemic male *ob*/*ob* mice and streptozotocin (STZ)-treated male Sprague Dawley rats; WT, C57BL/6 wild type mice; Ctrl, vehicle-treated control rats; * *p* < 0.05.

While large-scale transcriptome analyses have undeniably contributed to the discovery of ectopically expressed olfactory receptors and their cellular functions, these methodologies are not particularly adapted to the detection and quantification of lowly expressed genes [69,70]. Considering that GPCRs are usually expressed at low levels, our team took advantage of the broad dynamic range and low quantification limits of quantitative real-time PCR (qPCR) to screen human left ventricular tissue for the expression of 372 genes encoding ORs. Compared with bulk RNA sequencing, the use of qPCR arrays allowed for the identification of 38% more ORs expressed above the set detection threshold. By comparing expression patterns between normal individuals and patients diagnosed with non-ischemic cardiomyopathy, we were also able to demonstrate a dysregulated expression pattern for 38 ORs, including 18 ORs that were expressed in cardiomyocytes [71]. Interestingly, changes in the cardiac OR expression profile were sufficient to distinguish normal and diseased subjects, thus suggesting that alterations in cardiac OR signaling and functions may be of some relevance to the pathogenesis of heart failure (Figure 2).

While the above-mentioned recent studies have had a particular focus on ORs expressed in the cardiomyocytes, non-myocyte cell populations from the heart most likely contribute to the regulation of cardiac structure and function through ectopic OR signaling. Indeed, functional ORs have been described in other cell types abundantly found in the heart including fibroblasts, endothelial cells, vascular smooth muscle cells, and macrophages [72,73,74,75]. We summarize some of these reported effects below.

### 3.2. Regulation of Vascular Function

The olfactory receptor OR10J5 (Olfr16) has been shown to be expressed both in the human aorta and coronary arteries. Its reportedly high expression in human umbilical vein endothelial cells (HUVECs) further pointed toward a potential function for this OR in the vascular endothelium [73]. Exogenous ligands for OR10J5 include hydroxymethylpentylcyclohexenecarboxaldehyde (also known as lyral) and α-cedrene, respectively, a synthetic fragrance and a sesquiterpene derived from the essential oil of cedar [76,77]. The treatment of HUVEC with lyral led to OR10J5-dependent phosphorylation of the Akt kinase that was triggered by a rise in intracellular Ca^2+^ levels, as well as increased phosphorylation of extracellular signal-regulated kinase (ERK). Those signaling events were accompanied by increased migration of HUVECs in vitro and enhanced blood vessel formation in the mouse in vivo, thus establishing OR10J5 as a novel regulator of angiogenesis [73].

The olfactory receptor OR51E2 (Olfr78) in mice has been found to be expressed in the smooth muscle cells of the small resistance vessels of the heart, diaphragm, skeletal muscle, and skin. The activation of OR51E2 in these cells by propionate, a ligand that naturally occurs from the metabolic activity of the gut microbiota, is believed to be part of a counterregulatory mechanism aimed at mitigating the powerful hypotensive effect of the short-chain fatty acid mediated by the activation of other GPCRs such as Gpr41 [41,78]. The molecular activation of OR51E2 by propionate involves a rotation of the sixth transmembrane domain accompanied by the displacement of the third extracellular loop within the receptor [79]. Although this mechanism does not seem to play a role in the regulation of baseline blood pressure, the predominant expression of OR51E2 in arteriolar smooth muscle cells suggests a participation in the fine-tuning of the blood supply, especially to striated muscles. Such a mechanism may be useful as a cross-organ communication system to optimize the absorption of nutrients into the bloodstream [80,81].

More recently, targeting the activation of an ectopic OR signal transduction pathway has been proposed as a novel therapeutic strategy to tackle antiplatelet medication resistance and the detrimental progression of abdominal aortic aneurysm (AAA). Specifically, the increased expression of the olfactory receptor OR2L13 (Olfr166; Olfr168) was identified as a feature of platelets that are biomechanically activated by an increase in blood flow turbulence in AAA patients. Knocking out *Olfr168* increased both the rate of aortic growth and the incidence of aortic rupture in a mouse model of AAA, while treatment with the terpene (−)-carvone, an exogenous agonist of OR2L13, attenuated AAA growth in a manner that paralleled that of aspirin-based therapies [82].

### 3.3. Regulation of Inflammatory Processes

Inflammatory processes play a key role in the development and progression outcomes of many cardiovascular and renal diseases [83,84]. Chronic inflammation is also regarded as a central etiological mechanism for cardiometabolic diseases [85]. Therefore, any modulation of inflammatory cell functions through ectopic OR signaling is very likely to affect the cardiovascular–kidney–metabolic axis. While we still know very little about the extent of the repertoire of OR genes that are functionally expressed in leukocytes, it is noteworthy that several ORs were recently found to be highly expressed in acute myeloid leukemia (AML) cells in a pattern that could predict the prognosis of patients [86]. Moreover, activation of the olfactory receptor OR2AT4 (Olfr520) on leukemia cells with the synthetic agonist sandalore has been shown to inhibit cell proliferation and promote apoptosis through Ca^2+^-dependent activation of Erk1/2 [87]. There is also growing evidence that ORs promote or modulate the inflammatory response by macrophages, which was the topic of a recent review [74].

The first clue on the potential role of odorants in the modulation of macrophage function came from the study of mouse airway and pulmonary macrophages by Li and colleagues [88]. Using microarray analysis followed by qPCR validation, the investigators demonstrated that treatment with a combination of the immunogenic stimulators interferon-γ (IFN-γ) and lipopolysaccharide (LPS) increased the expression of eight ORs 50- to 250-fold in those cells. They further demonstrated that exposure of pulmonary macrophages to odorants, such as octanal, amyl acetate, or diaminopimelic acid, induced monocyte chemoattractant protein-1 (MCP-1) expression and enhanced cell migration under IFN-γ and LPS stimulation. Because octanal failed to influence the polarization and phagocytic activity of macrophages, and because the odorants tested may naturally originate from bacterial metabolic activity, the investigators proposed that this ectopic OR signaling in macrophages may serve as an alternative pathogen recognition pathway [88].

Despite the absence of any noticeable cell polarizing effect of the odorants tested in the initial study, Vadevoo and colleagues subsequently decided to investigate a potential link between OR51E2 signaling in macrophages and cancer progression [89]. Their hypothesis was based on previous findings that lactate activates OR51E2 in other cell types [50,90] and that increased lactate levels in the tumor microenvironment are a well-known metabolic signal for tumor-associated macrophage M2 polarization, thereby promoting tumor growth through increased angiogenesis and immunosuppression [91,92]. After initially confirming the expression of OR51E2 in macrophages, the investigators demonstrated the dependency of lactate-mediated M2 polarization on the presence of this OR at the plasma membrane. They also showed the effect is mediated by the interaction of OR51E2 with Gpr132, which is another GPCR and key macrophage sensor of rising lactate levels, and results in enhanced tumor progression and metastasis in mice [89]. The findings may have important implications in terms of managing the prognosis of individuals suffering from heart disease, not only in association with cancer detection and treatment but also in the setting of other cardiac conditions where the timing of macrophage polarization strongly influences functional outcomes such as during myocardial infarction [93,94,95]. Interestingly, myocardial ischemia triggers a metabolic switch within cardiomyocytes toward glycolysis and away from oxidative phosphorylation, which also creates a lactate-rich environment for both resident tissue macrophages and infiltrating bone marrow-derived monocytes recruited at the infarct zone following a heart attack [96,97,98]. Whether OR51E2 similarly plays a role in the polarization of macrophages during alteration of the cardiac metabolic environment by ischemia remains to be investigated.

In the vascular wall, multiple macrophage subsets play various defining roles in the development and progression of atherosclerotic lesions. One such role is the uptake and intracellular accumulation of lipoproteins that give birth to foam cells, which then promote both the growth and instability of atherosclerotic plaque via the release of pro-inflammatory cytokines and cell death-mediated extension of the necrotic core, respectively [99]. One particular subset of macrophages, which harbors a more aggressive inflammatory and pro-atherogenic gene expression signature, was also found to express a cluster of OR genes and OR-associated signal transduction proteins at higher levels [100]. A follow-up study identified OR6A2 (Olfr2) as a mediator of NLR family pyrin domain containing 3 (NLRP3) inflammasome activation and interleukin-1 (IL-1) secretion in both human and mouse macrophages. The aldehyde octanal, which can be generated in vivo from the peroxidation of dietary lipids, was found to accumulate at ligand-activating concentrations in atherosclerotic aorta. Treatment of *Apoe^−/−^* mice with octanal increased, while the OR6A2 antagonist citral decreased, atherosclerotic plaque lesion formation and inflammation caused by the consumption of a Western diet. Lastly, the specificity of OR–octanal signaling was confirmed by demonstrating the loss of the pro-atherosclerotic phenotype in *Ldlr^−/−^* mice that had been lethally irradiated and transplanted with bone marrow from *Olfr2^−/−^* or *Rtp1/2^dKO^* mice [101]. These data are a powerful illustration of how ectopic OR signaling may eventually be pharmacologically targeted for the treatment, prevention, and even reversal of life-threatening cardiovascular conditions (Figure 1).

## 4. Olfactory Receptors in the Kidney

### 4.1. Regulation of Blood Pressure

**Figure 3:**
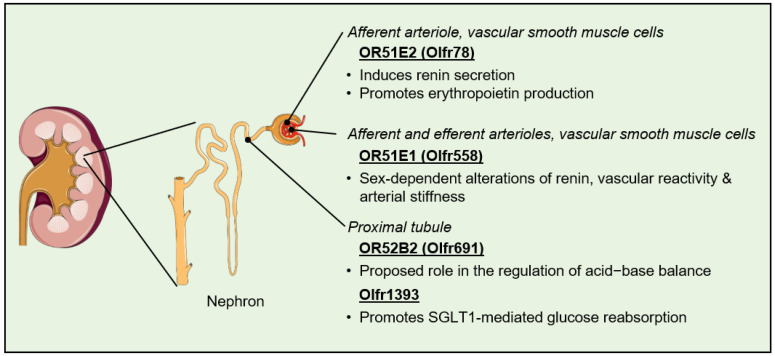
Biological effects of ectopic olfactory receptors expressed in the kidney. SGLT-1, sodium-glucose cotransporter 1.

Membrane receptors, and particularly GPCRs, are essential to the kidney’s ability to sense a variety of chemical cues from the bloodstream in order to maintain homeostasis of the body’s extracellular fluids and optimal blood pressure. The >700 GPCRs that have been shown to be expressed at significant levels within the different parts of the nephron include several dozens of ORs [102,103]. The first evidence for the existence of a functional OR signaling pathway within the kidney came in 2009 when Pluznick and colleagues immunolocalized G_olf_ and AC3 at the epithelial cells of the distal tubule and in the macula densa of murine nephrons. In addition to confirming the renal expression of six ORs by qPCR, the investigators were also able to demonstrate that the loss of AC3 resulted in the mouse kidneys being unable to properly regulate the glomerular filtration rate and plasma renin [51]. They subsequently demonstrated that among the six ORs identified, Olfr78 promoted the secretion of renin from the juxtaglomerular cells upon binding to its short-chain fatty acid ligand propionate. This mechanism, in conjunction with the direct antagonization of the hypotensive effects of Gpr41 in small resistance vessels, is proposed to contribute to the modulation of blood pressure by the gut microbiota [41,78,81]. While not related to its blood pressure regulatory effects, it is worth mentioning that renal Olfr78 may also contribute to the cardiorespiratory response to hypoxia, possibly via the stimulation of erythropoietin production [104]. The olfactory receptor OR51E1 and the classical components of OR intracellular signaling have also been found to be expressed in the human-derived, proximal tubular cell line HK-2. Exposure of those cells to the branched-chain saturated fatty acid isovaleric acid, an OR51E1 ligand, leads to an increase in cytosolic Ca^2+^ levels [105]. Interestingly, genome-wide association studies and the analysis of *Olfr558* knockout mice indicate that this OR plays an evolutionary conserved role in mediating sex differences in blood pressure through alterations in renin levels, vascular reactivity, and arterial stiffness [106,107]. Collectively, these studies shed light on renal OR signaling as a physiologically relevant contributor to blood pressure regulation by environmental and innate factors (Figure 3).

### 4.2. Regulation of Fibrosis and Kidney Stone Formation

While the participation of renal OR signaling in the pathophysiology of kidney diseases has not yet been established, a few studies have pointed out a correlation between altered OR expression, the activation of pro-fibrotic pathways, and the development of renal fibrosis leading to CKD [108,109]. The formation of kidney stones, which is a risk factor for CKD, has also been associated with dysregulated expression of several ORs in the renal papillary tips [110,111]. Studies specifically investigating the expression and function of ectopic ORs in fibroblasts of the cardiovascular and renal systems are still lacking. However, one study employing the Hs68 fibroblast cell line derived from human skin demonstrated that those cells express at least 10 different ORs, and among those ectopically expressed ORs, OR51B5 (Olfr65) is critical to the maintenance of gene clusters associated with cell survival and collagen synthesis [75]. Fibrosis in CKD is a progressive pathogenic process that not only impairs kidney function but is also closely interlinked with hypertension and causes severe myocardial fibrosis and contractile dysfunction as well [112,113,114]. Interestingly, two independently conducted studies have shown that the manipulation of the gut microbiome in rat models of CKD, either through the administration of certain medicinal plant extracts or via treatment with resveratrol-synthesized derivatives, altered the profile of circulating short-chain fatty acids and decreased the renal expression of OR51E2, which was associated in both cases with a decrease in blood pressure and the amelioration of kidney function [115,116]. Clearly, the potential contribution of ectopic ORs in the development of fibrotic diseases affecting the cardiovascular–renal axis is an intriguing concept that deserves further investigation.

### 4.3. Regulation of Filtration, Reabsorption, and Secretion

The olfactory receptor Olfr691 (OR52B2) was identified by the Pluznick team while screening for novel renal sensory receptors. Using a heterologous expression system, the research team also characterized thirteen new short- and medium-chain fatty acids as Olfr691 agonists, in addition to the previously reported ligands valeric and isovaleric acids [117]. Given the localization of Olfr691 transcripts in the proximal tubule, a possible role for this OR in the mitigation of metabolic acidosis through the alteration of bicarbonate secretion has been proposed [103,117]. Another OR ectopically expressed in the murine proximal tubule, namely, Olfr1393 (Human ortholog unknown), has been shown to play a role in the regulation of glucose reabsorption. Indeed, mice lacking Olfr1393 exhibit glycosuria that is associated with improved glucose tolerance and protection from high-fat diet-induced hyperfiltration [118,119]. This phenotype appears to be linked to the decreased localization of sodium–glucose co-transporter 1 (SGLT1) at the luminal membrane subsequent to the loss of the direct interaction between the two proteins [118]. While such functions remain to be confirmed, and the molecular mechanisms involved need to be clearly elucidated, these findings have revealed new biological pathways that may potentially allow the modulation of renal physiology in disease states (Figure 3). The modulation of glucose reabsorption is a particularly exciting area, considering the proven benefits of SGLT2 inhibition for the care of patients suffering from heart failure with either reduced or preserved ejection fraction [120,121].

## 5. Ectopic Olfactory Receptors in the Control of Metabolic Homeostasis

### 5.1. Regulation of Pancreatic Hormone Secretion

**Figure 4:**
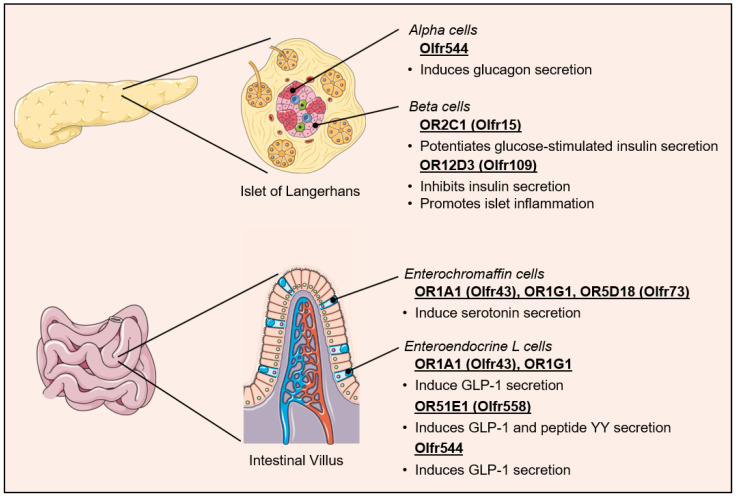
Regulation of pancreatic hormone and incretin secretion by ectopic olfactory receptors. GLP-1, glucagon-like peptide-1.

As illustrated above for Olfr1393 in the kidney, ectopic OR signaling may impact cardiometabolic health through the direct regulation of systemic metabolism. One such mechanism involves modulating the secretion of incretins and pancreatic hormones that control glucose and lipid utilization in metabolically active tissues (Figure 4). By combining gene expression analysis and immunofluorescence of mouse tissue and cultured cells, Kang and colleagues reported the expression of several OR genes and OR signaling proteins G_olf_ and AC3 in the α-cells of the islets of Langerhans [122]. The receptive range of one of the ORs identified, Olfr544 (Human ortholog unknown), had previously been shown to encompass 8- to 12-carbon chain length mono and dicarboxylic acids [31,123]. Treatment of the α-cells with the nine-carbon chain length dicarboxylate azelaic acid led to a dose- and time-dependent increase in intracellular Ca^2+^ levels and glucagon secretion, an effect that was abrogated by siRNA-mediated inhibition of Olfr544 [122].

Using DNA microarray analysis of isolated mouse pancreatic islets and MIN6 cells, the Katagiri group similarly demonstrated that at least 47 ORs are expressed at the mRNA level in β-cells. Among those, both Olfr15 (OR2C1) and Olfr821 (OR6C74) were immunodetected in almost all β-cells of the pancreatic islets. Furthermore, the medium-chain fatty acid octanoate, which had previously been shown to interact with OR2C1, potentiated glucose-stimulated insulin secretion from those same isolated pancreatic islets and MIN6 cells, an effect that was lost upon siRNA-mediated inhibition of Olfr15 [124,125]. Similar findings were made by Leem and colleagues, and both research teams concluded the effect to be dependent on the activation of a phospholipase C (PLC)-IP3 signaling pathway [124,126]. Leem and colleagues further demonstrated that the long-term stimulation of OR2C1 with octanoate resulted in the up-regulation of glucokinase and enhanced glucose uptake through the IP3-CaMKK/CaMKIV pathway [126]. These findings provided a molecular explanation for the long-known insulinotropic effect of octanoate [127]. Interestingly, the receptive range for OR2C1 is notoriously wide and, besides aliphatic compounds, includes saturated and unsaturated cyclic compounds, nitrotoluenes, alcohols, aldehydes, and terpenes, among other compounds [128,129,130]. Thus, the results highlight the critical function of ectopic ORs in the pancreas as a chemo-sensing system used to tune the release of hormones according to the composition and concentrations of not only nutrients but also chemicals and toxins circulating in the bloodstream.

### 5.2. Regulation of Incretin Secretion

The cells lining the gastrointestinal tract are continuously exposed to molecules originating from dietary and microbial sources, making them ideally located to sense and integrate signals for the control of metabolic homeostasis. Functional olfactory receptors have been identified on the enterochromaffin and enteroendocrine L cells of the intestine [131]. In 2007, Braun and colleagues were the first to report the expression of four ORs in human enterochromaffin cells as follows: OR1A1 (Olfr43; Olfr403), OR1G1 (Mouse ortholog unknown), OR1E3 (now classified as a pseudogene), and OR5D18 (Olfr73). Treatment of enterochromaffin cells with various odorants known to bind those receptors (including eugenol, thymol, bourgeonal, and helional) elicited an increase in intracellular Ca^2+^ levels and the secretion of serotonin [132]. In addition to regulating gut motility and the absorption of nutrients, serotonin may play a direct role in the stimulation of glucagon-like peptide-1 (GLP-1) release by enteroendocrine L cells, and it may even stimulate the release of insulin in the β-cells of the pancreatic islets [133,134].

A decade later, Kim and colleagues also identified the presence of functional OR1A1 (Olfr 43) and OR1G1 receptors in enteroendocrine L cells. The identification was based on the ability of geraniol and citronellal, two known agonists of the receptors, to stimulate the secretion of GLP-1 from isolated mouse intestinal tissue and a human-derived enteroendocrine L cell line [135]. Using an elegant RNA silencing approach, the investigators confirmed the involvement of a G_olf_-cAMP-mediated increase in intracellular Ca^2+^ levels in the enteroendocrine L cell OR signaling. Lastly, the physiological relevance of this novel mechanism of GLP-1 secretion was established in *db*/*db* mice, where geraniol treatment improved insulin secretion and blood glucose disposal in response to an oral glucose tolerance test, an effect that was negated by pre-injection of the GLP-1 receptor antagonist Ex9-39 [135]. As already noted for OR2C1 in β-cells, the repertoire of odorants known to activate OR1A1 is relatively large, thus supporting the notion that GLP-1 secretion may similarly be finely regulated by a wide range of physiological and pathophysiological conditions [136,137,138]. The complexity of GLP-1 regulation by odorants is further illustrated by the recent discovery of additional ORs modulating its secretion. The olfactory receptor OR51E1 is also expressed in enteroendocrine L cells of rodents, pigs, and humans, and stimulation of these cells with nonanoic acid leads to an OR51E1-dependent increase in GLP-1 and peptide YY secretion [139,140]. The olfactory receptor Olfr544 was found to be expressed in the mouse small intestine and colon, and in vivo mouse treatment as well as in vitro stimulation of the murine GLUTag cell line with azelaic acid both induce GLP-1 release [141]. Whether the secretion of the glucose-dependent insulinotropic polypeptide (GIP) by the enteroendocrine K cells is similarly regulated by ectopically expressed ORs remains to be determined.

### 5.3. Regulation of Metabolic Tissue Function

**Figure 5:**
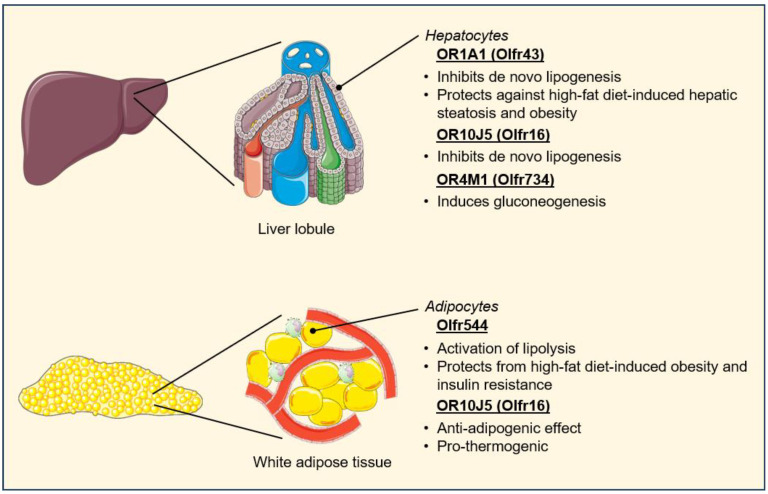
Regulation of hepatic and white adipose tissue metabolic processes by ectopic olfactory receptors.

Besides the gut and the pancreas, ectopic ORs have been detected in other organs and tissues that are critical to the regulation of energy intake and utilization, including the liver, adipose tissue, and skeletal muscle (Figure 5). While the number of OR genes expressed in the liver appears to be relatively low, the contribution of hepatic ORs to the regulation of glucose and lipid metabolism is well established [53,67]. The Lee lab first reported that the olfactory receptor OR1A1 is expressed at the plasma membrane of HepG2 hepatocytes and that treatment of those cells with the OR1A1 agonist (−)-carvone led to the down-regulation of the lipogenic transcription factor peroxisome proliferator-activated receptor (PPAR)-γ through a cAMP-PKA-cAMP response element-binding protein (CREB) signaling pathway, thus resulting in a decrease in intracellular triglyceride levels [142]. In a follow-up study, the investigators demonstrated that in vivo stimulation of the OR1A1 ortholog Olfr43 resulted in the activation of a similar pathway in mouse livers, which was accompanied by a reduction in hepatic steatosis and adiposity under high-fat feeding conditions [143]. The functionality of the olfactory receptor OR10J5 (Olfr16) has also been investigated in HepG2 cells. Following the characterization of α-cedrene as a natural ligand of OR10J5 in olfactory sensory neurons, treatment of the human hepatocytes with the sesquiterpene was found to reduce intracellular triglyceride accumulation through molecular mechanisms similar to those regulated by OR1A1 [77]. Lastly, the identification of hepatic OR4M1 (Olfr734) as the receptor for the adipokine asprosin is another recent example of how ectopic ORs regulate metabolic homeostasis via inter-organ communication. Asprosin is a fasting-induced protein hormone that was initially found to activate hepatocyte glucose production through a cAMP-PKA mechanism [144]. While asprosin signaling was also shown at the time to require a G-protein, it was a few years later that experimentations with *Olfr734* knockout mice revealed hepatic OR4M1 to be the receptor mediating the gluconeogenic effects of this hormone [145].

The olfactory receptor Olfr544, previously mentioned for its roles in the secretion of glucagon and GLP-1, has also been found to be expressed in the liver and adipose tissue of mice. Using 3T3-L1 adipocytes, the Lee lab reported that the Olfr544 agonist azelaic acid leads to PKA-mediated phosphorylation and activation of hormone-sensitive lipase (HSL), the rate-limiting enzyme in lipolysis. In vivo, this mechanism translates into reduced adiposity and improved insulin sensitivity for mice fed a high-fat diet, although Olfr544-mediated activation of mitochondrial biogenesis in skeletal muscle should also probably be considered as a determining factor in this phenotype [146,147]. In a separate study, 3T3-L1 cells were used to investigate the functionality of OR10J5 signaling in white adipose tissue. The knockdown of this OR was found to increase intracellular lipid accumulation and to abrogate the lipid-lowering effect of α-cedrene, thus illustrating the proposed anti-adipogenic and thermogenic functions of adipocyte OR10J5 signaling through up-regulation of the transcription factors CCAAT/enhancer-binding protein (CEBP)-α and peroxisome proliferator-activated receptor-γ coactivator 1(PGC1)-α, respectively [148].

### 5.4. Regulation of Cardiovascular–Renal Metabolic Health

Systemic metabolic disorders, including metabolic syndrome, obesity, and diabetes, act as initiating events in the pathogenesis of the cardiovascular–kidney–metabolic syndrome [10,11]. Single nucleotide polymorphisms in several dozens of OR genes have been linked to the increased occurrence of such metabolic disorders [149,150]. It is now well recognized that by altering the sense of smell, dysfunction of olfactory signaling in the nasal epithelium can directly predispose to poor eating habits and body weight gain [150]. On the other hand, the role played by altered ectopic OR signaling in the impairment of systemic metabolic homeostasis is much less well appreciated. Expression of the gene encoding the olfactory receptor Olfr109 (OR12D3) in mouse pancreatic β-cells was recently found to be up-regulated with obesity and diabetes and directly linked to the impairment of glucose homeostasis. It was further determined that Olfr109 acts as a receptor for denatured insulin and insulin-derived peptides, thereby triggering molecular pathways associated with the inhibition of insulin secretion and increased inflammatory processes in the pancreatic islets (Figure 4). In addition, the pharmacological blockade of Olfr109 with pepducins successfully improved glucose metabolism during obesity and diabetes [151]. On another note, and although the potential modulation of renal Olfr1393 expression with obesity and diabetes remains to be investigated, it is noteworthy that the deletion of the receptor in mice leads to a significant improvement in hyperglycemia and glucose tolerance in response to streptozotocin-induced type 1 diabetes [152]. We also found evidence for altered cardiac expression of the olfactory receptor OR51E2 in rodent models of impaired glucose tolerance and diabetes, although here again, the functional meaning of the dysregulation remains to be established (Figure 2). These observations warrant further research into the role that altered ectopic OR signaling may play in the development of cardiovascular–renal disorders associated with metabolic diseases.

## 6. Conclusions

In a little over a decade, ectopic ORs have established themselves as critical actors in the regulation of mammalian physiological processes. Olfactory receptor functionality has now been demonstrated in most cell types composing the cardiovascular system. Olfactory receptors are also abundantly expressed in the kidney and in major metabolic tissues, with mounting evidence that they play a fundamental role in cross-organ communication through the recognition of circulating metabolites and even peptide and protein hormones. The growing identification of ectopic OR ligands and signaling pathways modulated in these tissues will likely provide a deeper understanding of how environmental factors influence cardiovascular–kidney–metabolic health. In addition to pointing to novel mechanisms by which unhealthy dietary habits promote cardiovascular disease, ectopic OR functions may also provide valuable insight into the relationships existing between exposure to pollutants and toxic chemicals and disruption of the mammalian hormonal and metabolic milieu. Beyond the cardiovascular–kidney–metabolic syndrome, the pathophysiological implications of ectopic OR signaling in human-acquired diseases are enormous and may also encompass neurodegenerative disorders and several types of cancer [153,154]. Generally low expression levels in non-olfactory tissues, persisting difficulties in identifying endogenous ligands, and the lack of a high-quality repertoire of specific OR antibodies still represent significant barriers to the characterization of ectopic ORs. Nonetheless, there is little doubt that future research in this area will eventually lead to the identification of novel biomarkers and therapeutic targets for an improved management of cardiovascular disease.

## Data Availability

Most of the data supporting the views and conclusions expressed in this review article were derived from previously published articles and are available in the public domain. Details of the specific studies included in this review article can be found in the referenced articles. Data presented in Figure 2 were generated by the authors via reanalysis of samples from their previously published studies, and the raw data are available upon request by contacting the corresponding author of this review article.
